# Metabolic comorbidities among patients with peripheral artery disease: a national population-based study

**DOI:** 10.1093/geroni/igab046.2346

**Published:** 2021-12-17

**Authors:** Gi Wook Ryu, Mona Choi, Young Shin Park

**Affiliations:** 1 Namseoul university, Cheonan, Ch'ungch'ong-namdo, Republic of Korea; 2 Yonsei University, Seoul, Seoul-t'ukpyolsi, Republic of Korea

## Abstract

Peripheral artery disease (PAD) is a chronic disease which is associated with old age. PAD was known as an age-related chronic condition. Metabolic comorbid conditions which include hypertension, diabetes, and hyperlipidemia can have negative impacts on blood vessels aggravating PAD in elderly patients. Therefore, metabolic comorbidities need be considered in order to develop intervention for patients with PAD. The aim of this study is to find the characteristics of PAD patients with metabolic comorbidities. This is a retrospective study that used the national claim data of South Korea from 2009 to 2018. The inclusion criteria were adults (20+) and patients diagnosed with PAD as a primary or secondary diagnosis from 2011 to 2017. The frequency of hypertension, hyperlipidemia, diabetes, and metabolic comorbidities in PAD patients was examined. In addition, the difference in the number of metabolic comorbidities according to sex was identified using the chi-squared test. Among the total PAD adult patients(n=8,478,876), the number of elderly patients over 60 years old was 4,124,592(48.7%). Among the total patients, PAD patients with hypertension were the most common at 958,329 (11.30%). Sex was significantly related to having metabolic comorbidities and women showed higher proportion of metabolic comorbidities compared to men (χ2 =5.02, p<.001). As the frequency of PAD patients with hypertension were the highest, it is necessary to develop a health management program that considers metabolic comorbidities, especially hypertension, in order to manage PAD disease. In addition, there is a need for special interest in intervening metabolic conditions of female patients with PAD.

